# Prevalence and Species Diversity of Spotted Fever Group Rickettsiae in Ixodid Ticks Collected in Northwest Russia

**DOI:** 10.3390/tropicalmed11070179

**Published:** 2026-06-27

**Authors:** Islam Karmokov, Olga Freylikhman, Regina Baimova, Daria Grechishkina, Gelena Lunina, Ivan Lyzenko, Ekaterina Riabiko, Tatiana Arbuzova, Anastasiia Bachevskaia, Edward Ramsay, Erik Khalilov, Karina Kukleva, Lyubov Bespyatova, Sergey Bugmyrin, Maxim Petrov, Olga Neverova, Ksenia Titarchuk, Vera Agasoi, Nikolai Kalinin, Olga Vorobyeva, Olga Mikheenko, Tatiana Iakimenko, Inna Druzhinina, Olga Matina, Daria Monastyrskaya-Nuzhina, Anna Smirnova, Nikolay Tokarevich

**Affiliations:** 1 Saint Petersburg Pasteur Institute, Saint Petersburg 197101, Russia; freilikhman@pasteurorg.ru (O.F.); baimova@pasteurorg.ru (R.B.); grechishkina@pasteurorg.ru (D.G.); lunina@pasteurorg.ru (G.L.); lyzenko@pasteurorg.ru (I.L.); riabiko@pasteurorg.ru (E.R.); arbuzowa95@yandex.ru (T.A.); nastyabachevskaya@gmail.com (A.B.); zoonoses@mail.ru (N.T.); 2 North-West Plague Control Station, Saint Petersburg 198035, Russia; erik.khalilov@yandex.ru; 3 Saint Petersburg State Pediatric Medical University, Faculty of General Medicine, Saint Petersburg 194100, Russia; karina5476875@yandex.ru; 4 Institute of Biology of Karelian Research Centre RAS, Petrozavodsk 185910, Republic of Karelia, Russia; gamasina@mail.ru (L.B.); sbugmyr@mail.ru (S.B.); 5 Center of Hygiene and Epidemiology in the Arkhangelsk Region and the Nenets Autonomous Okrug, Arkhangelsk 163001, Arkhangelsk Region, Russia; petrov_ms@fbuz29.rospotrebnadzor.ru (M.P.); konstnev@yandex.ru (O.N.); k.titarchuk@mail.ru (K.T.); 6 Pskov State University, Department of General Biology and Biomedicine, Pskov 180000, Pskov Region, Russia; agasoi_87@mail.ru; 7 Center for Hygiene and Epidemiology in the Pskov Region, Pskov 180000, Pskov Region, Russia; info@60cgie.ru (N.K.); pskovfgus@yandex.ru (O.V.); 8 Center for Hygiene and Epidemiology in the Kaliningrad Region, Kaliningrad 236016, Kaliningrad Region, Russia; miheenko_op@mail.ru (O.M.); kononenkots94@mail.ru (T.I.); 9 Center for Hygiene and Epidemiology in the Novgorod Region, Novgorod 173002, Novgorod Region, Russia; innadruzinina06@gmai.com (I.D.); paraz_mon@cgevnov.ru (O.M.); nuzhina97@inbox.ru (D.M.-N.); 10 Center for Hygiene and Epidemiology in the Vologda Region, Vologda 160012, Vologda Region, Russia; ansm30@mail.ru

**Keywords:** Ixodidae, Northwest Russia, PCR, prevalence, spotted fever group Rickettsiae, vegetation

## Abstract

*Rickettsia* spp. are ubiquitous in nature and capable of causing diseases of varying severity. The most extensive group comprises the spotted fever group (SFG) Rickettsiae, the members of which are predominantly transmitted by ticks. The expansion of tick habitats observed in recent decades poses an increasing threat of dissemination of tick-borne infections into regions previously considered non-endemic. The aim of this study was to determine the prevalence of SFG Rickettsiae in ixodid ticks collected in Northwest Russia and to characterize the species diversity of these pathogens within the study area. Questing adult ixodid ticks (*n* = 4566) were collected from eight regions of Northwest Russia (Arkhangelsk, Kaliningrad, Leningrad, Novgorod, Pskov and Vologda Regions, as well as the Republic of Karelia and St. Petersburg) in 2023 to 2025 (from April to September). The species composition included *Ixodes ricinus* (*n* = 1683), *Ixodes persulcatus* (*n* = 2404), and *Dermacentor reticulatus* (*n* = 479). Genomic DNA was extracted from individual ticks and screened for SFG Rickettsiae using real-time PCR, followed by conventional PCR targeting the *gltA*, *ompA*, *ompB*, and *sca4* (gene D) genes. Nucleotide sequences obtained for a subset of positive samples for the various genes were analyzed. The overall prevalence of SFG Rickettsiae was 12.6% (95% CI: 11.7–13.6). Circulation of the following species was detected: *Rickettsia helvetica*, *Rickettsia conorii* subsp. *raoultii*, *Candidatus* Rickettsia tarasevichiae, *Rickettsia monacensis*, and *Rickettsia felis*. The findings indicate considerable species diversity of SFG Rickettsiae in natural foci of Northwest Russia. *Rickettsia monacensis* was detected in ixodid ticks within the study area for the first time, and *R. felis* was identified in Russia for the first time.

## 1. Introduction

*Rickettsia* spp. (order *Rickettsiales*, family *Rickettsiaceae*) are Gram-negative, obligate intracellular α-proteobacteria primarily transmitted by arthropods (ticks, fleas, lice, mosquitoes, and others), many of which are pathogenic to humans and animals. *Rickettsia* spp. are ubiquitous in nature and are capable of causing diseases of varying severity, varying from mild (e.g., rickettsiosis caused by *R. helvetica*) to severe, such as epidemic typhus and Rocky Mountain spotted fever [1].

According to current classification based on pan-genome meta-analysis of its members, the genus *Rickettsia* is subdivided into several groups. The typhus group (TG) includes *R. prowazekii* and *R. typhi*. There are two spotted fever groups (SFGs): SFG I (*R. rickettsii*, *R. slovaca*, *R. conorii*, *R. sibirica*, *R. conorii* subsp. *raoultii*, *R. monacensis*, *R. africae*, and others); and SFG II (*R. felis*, *R. akari*, *R. australis*, and others). In addition, there is an *R. canadensis* group (Canadensis group, CG) and an *R. bellii* group (Bellii group, BG) [2]. Although candidate species *Candidatus* R. tarasevichiae is phylogenetically assigned to the CG based on its genetic characteristics [3,4], it is considered alongside SFG Rickettsiae in this study [1]. This approach is quite common and is justified by their shared reservoir, similar clinical picture, and serological cross reactions with members of the SFG.

The most extensive group comprises SFG I Rickettsiae, the members of which are predominantly transmitted by ticks. As a rule, specific tick species serve as vectors for specific SFG Rickettsiae species. Consequently, rickettsial diseases caused by these *Rickettsia* species may be considered endemic within the geographic range of their respective tick vectors [5]. The expansion of ticks into new regions may lead to the emergence of rickettsial diseases in previously non-endemic regions [6]. Over recent decades, certain tick species have expanded their geographic areas, primarily due to anthropogenic environmental impacts and climate change [5]. These changes pose an increasing threat of dissemination not only of tick-borne rickettsioses, but also of other tick-borne infections.

Many SFG Rickettsiae species are considered capable of transovarial transmission [7,8,9]. This suggests that ticks serve not only as vectors, but also as key reservoirs of *Rickettsia*, highlighting the importance of monitoring the prevalence of these pathogens in ticks as a critical component of epidemiological surveillance for tick-borne rickettsioses. The epidemiology of tick-borne rickettsial diseases depends on the geographic distribution and seasonal activity of tick vectors and their vertebrate hosts involved in pathogen transmission. Therefore, information on vector distribution, the frequency of occurrence and species diversity of SFG Rickettsiae in a given region, as well as the detection of novel pathogens in ticks is of considerable medical importance. The main epidemiologically significant tick species occurring in Northwest Russia are *Ixodes ricinus*, *Ixodes persulcatus*, and *Dermacentor reticulatus* [10,11]. These species are capable of acting as both vectors and reservoirs for a broad range of SFG Rickettsiae that cause tick-borne rickettsioses of varying severity [1]. These tick species feed on a variety of wild and domestic mammals, depending on developmental stage [12]. Larvae and nymphs feed on small (rodents, insectivores, hedgehogs) and medium-sized mammals, as well as on ground-feeding birds. Adults feed on large and medium-sized mammals, including wild and domestic ungulates (deer, wild boar, cattle, sheep), carnivores, and hares.

The aim of this study was to determine the prevalence of SFG Rickettsiae in ixodid ticks collected in Northwest Russia and to characterize the species diversity of these pathogens within the study area.

## 2. Materials and Methods

### 2.1. Tick Collection

**Figure 1:**
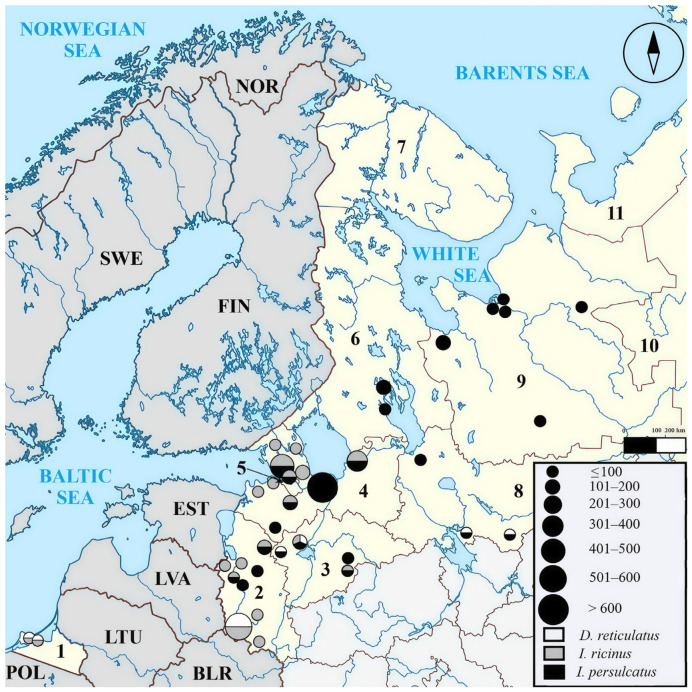
Tick collection sites. As per the figure legend, the size of each circle corresponds to the number of ticks collected within a given administrative region, whereas the shading indicates tick species composition. Abbreviations: BLR–Belarus, EST—Estonia, FIN—Finland, LTU—Lithuania, LVA—Latvia, NOR—Norway, POL—Poland, SWE—Sweden. Numbered regions: 1—Kaliningrad Region, 2—Pskov Region, 3—Novgorod Region, 4—Leningrad Region, 5—St. Petersburg, 6—the Republic of Karelia, 7—Murmansk Region, 8—Vologda Region, 9—Arkhangelsk Region, 10—the Komi Republic, 11—Nenets Autonomous Okrug. Note: tick collection was not performed in regions 7, 10, or 11.

During the period from 2023 to 2025, 4566 adult ixodid ticks were collected across 37 administrative regions within eight regions of Northwest Russia (Arkhangelsk, Kaliningrad, Leningrad, Novgorod, Pskov and Vologda Regions, as well as the Republic of Karelia and St. Petersburg) (Figure 1).

Questing ticks were collected from vegetation during their period of seasonal activity (from April to September) using a 1 × 0.6 m flannel flag. Attached ticks were carefully removed every 5 min using anatomical forceps and placed into 1.5 mL screw-cap test tubes, which were subsequently transported to the laboratory at +4 °C within 24 h for further analysis. Each tick was placed into its own tube labeled with information on the collection site and date. Ticks were identified to developmental stage, species, and sex morphologically under a stereomicroscope according to standard taxonomic keys [13,14]. After morphological identification, ticks were individually stored at −20 °C until nucleic acid extraction.

### 2.2. Homogenization and Nucleic Acid Extraction

Ticks were homogenized individually in 400 µL of sterile phosphate-buffered saline (PBS, pH 7.2–7.6, Sigma-Aldrich, St. Louis, MO, USA) containing sterile 4.5 mm diameter steel beads using a FastPrep-24™ mechanical homogenizer (MP Biomedicals, Irvine, CA, USA). Following centrifugation of the homogenates, a 100 µL aliquot of the supernatant was collected and stored at −80 °C until total nucleic acid (NA) extraction. Total NA extraction was carried out using the RIBO-prep RNA/DNA Extraction Kit (AmpliSens, Moscow, Russia) according to manufacturer instructions. The resulting NA samples were stored at −20 °C until further analysis.

### 2.3. Screening of Tick DNA for the Presence of SFG Rickettsiae Genetic Material

As an initial step, all the DNA samples obtained from the 4566 ticks were screened for the presence of *Rickettsia* spp. SFG genetic material. For this, the AmpliSens *Rickettsia* spp. SFG-FL kit (AmpliSens, Russia) was used according to manufacturer instructions. The kit targets the amplification and detection of an outer membrane protein gene (*ompB*) fragment. To prevent amplicon carryover contamination, this test system incorporates uracil-DNA glycosylase (UDG). Furthermore, an exogenous internal control and a negative extraction control were included in each total NA extraction procedure. Both negative and positive controls supplied with the reagent kit were included in each PCR run. Real-time PCR was performed on a CFX96 C1000 Touch™ thermal cycler (Bio-Rad, Hercules, CA, USA).

### 2.4. Sequencing and Phylogenetic Analysis

Following the screening phase, 577 tick samples that tested positive for SFG Rickettsiae DNA by real-time PCR were selected from a total of 4566 tick samples. For subsequent *Rickettsia* species identification, 250 tick samples with Ct values ≤35 were selected from this group. In the next phase, to determine the optimal target for *Rickettsia* species identification, fragments of the citrate synthase gene (*gltA*), outer membrane protein genes (*ompA*, *ompB*), and *sca4* (gene D) were sequenced for a subset of 80 samples from this group.

Conventional PCR amplification was performed using BioMaster HS-Taq PCR-Color (2×) master mix (Biolabmix, Novosibirsk, Russia). The reaction mixture (total volume 25 µL) contained 0.2 mM each of dNTPs, 1.25 U of DNA polymerase, and 0.4 µM each of oligonucleotide primers (GenTerra, Russia) with 5 µL (10–50 ng/µL) of sample DNA. Both negative and positive controls were included in each run: negative PCR control; a negative NA extraction control; and NAs extracted from *R. sibirica* (strain 1-K) and *R. conorii* (strain Conori) strains. The control strains were obtained from the *Rickettsia* collection, Laboratory of Zooanthroponosis (Saint Petersburg Pasteur Institute, Saint Petersburg, Russia).

PCR amplification and sequencing were performed using the forward and reverse primers listed in Table A1. The following conditions were used: initial denaturation at 95 °C for 5 min (1 cycle); followed by 40 cycles of denaturation at 95 °C for 15 s, primer annealing for 20 s (annealing temperatures varied depending on the primers used), and elongation at 72 °C for 50 s; with a final elongation at 72 °C for 5 min (1 cycle). PCR amplification was performed using a GeneExplorer GE-96S thermal cycler (Bioer, Hangzhou, China). PCR products were visualized by electrophoresis (PowerPac Basic, Bio-Rad, USA, 120 V, 400 mA, 25 min) on a 1.5% agarose gel (Agarose Special, Low EEO for Molecular Biology, HiMedia, Mumbai, India) in 1× Tris-borate-EDTA (TBE) buffer (Evrogen, Moscow, Russia) stained with ethidium bromide, under UV illumination.

The amplification and sequencing efficiency for the *gltA* gene fragment reached 99%, whereas that for the other genes was lower. Based on these results and given that the *gltA* gene is the standard marker for identifying SFG Rickettsiae species, this gene was selected for further analyses.

Amplicon purification was performed using Agencourt AMPure XP magnetic beads (Beckman Coulter, Brea, CA, USA) according to manufacturer instructions. Sanger sequencing was carried out using the Onelife BD3 Cycle Sequencing Kit (One-Life Gene Technology, Shenzhen, China). Sequencing products were analyzed on a 3500xL Genetic Analyzer (Applied Biosystems, Thermo Fisher Scientific, Waltham, MA, USA). Sequence chromatograms were visualized and edited using UGENE software version 46.0 (Unipro, Russia). *Rickettsia* species were identified by comparison with sequences deposited in the NCBI GenBank database (https://www.ncbi.nlm.nih.gov/genbank/ (accessed during the period from 14 October 2024 to 17 March 2026)) using the BLAST (Basic Local Alignment Search Tool, version 2.17.0) algorithm (https://blast.ncbi.nlm.nih.gov/Blast.cgi (accessed during the period from 14 October 2024 to 17 March 2026)). Species-level identification was assigned based on 99–100% sequence identity with the corresponding reference sequences.

Phylogenetic relationships among *Rickettsia* isolates were inferred based on the analysis of *gltA* gene fragment sequences. Multiple sequence alignment was performed using MEGA 12 software. Based on the Akaike Information Criterion (AIC) implemented in the “Find Best DNA/Protein Model” function, the T93+G model (Tamura 3-parameter with gamma distribution) was selected as the optimal nucleotide substitution model. A phylogenetic tree was reconstructed using the maximum likelihood (ML) method in MEGA 12. Branch support was assessed by bootstrap analysis with 1000 pseudoreplicates.

Analysis included nucleotide sequences generated in this study, which were grouped according to sequence identity, tick host species, and collection locality. The phylogenetic tree was rooted using *gltA* fragment sequences of *Rickettsia* species belonging to the TG, BG, and CG. For comparative analysis, the genetically closest reference sequences retrieved from the NCBI GenBank database were included (Table A2).

### 2.5. Statistical Analysis

Prevalence is expressed as a percentage and was calculated with a 95% confidence interval (CI) for proportions using the exact Clopper–Pearson method. *p*-values were calculated using the two-tailed Fisher’s exact test, supplemented by the Mantel–Haenszel estimate of the common odds ratio (OR), and were adjusted for multiple comparisons using Holm’s correction. *p*-values ≤ 0.05 were considered statistically significant. All calculations were performed using the Python 3.13.9 software.

## 3. Results

The collected ticks belonged to three species: *Ixodes ricinus* (Linnaeus, 1758) (37%), *Ixodes persulcatus* (Schulze, 1930) (53%), and *Dermacentor reticulatus* (Fabricius, 1794) (10%). The female-to-male ratio was 1:1. The results of tick screening for the presence of SFG Rickettsiae DNA are presented in Table 1.

The SFG Rickettsiae prevalence in *I. persulcatus* was significantly lower than that found in *D. reticulatus* and *I. ricinus* (*p* < 0.001; OR = 11.4 [95% CI: 8.4–15.5] and 10.8 [95% CI: 8.3–14.0], respectively). No statistically significant difference in prevalence was detected between *D. reticulatus* and *I. ricinus* (*p* > 0.05; OR = 1.2 [95% CI: 0.9–1.6]). Prevalence among female ticks was significantly higher than that among males (*p* < 0.001; OR = 1.5 [95% CI: 1.2–1.8]).

**Figure 2:**
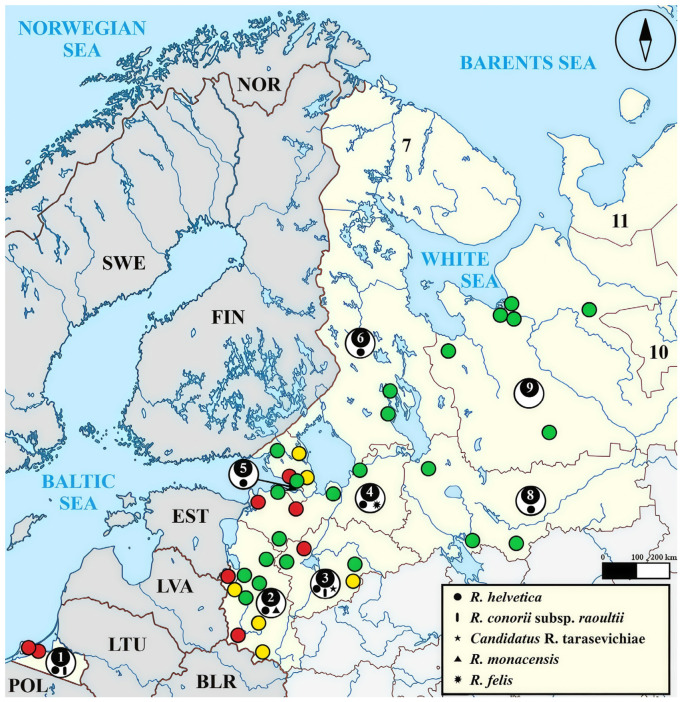
Stratification of administrative regions according to the level of SFG Rickettsiae prevalence in ticks. Regions with an SFG Rickettsiae prevalence of >13.6% are indicated in red, those with 11.7–13.6% in yellow, and those with <11.7% in green. As per the legend, distinct geometric symbols denote the SFG Rickettsiae species detected within each respective region of Northwest Russia. Abbreviations: BLR—Belarus, EST—Estonia, FIN—Finland, LTU—Lithuania, LVA—Latvia, NOR—Norway, POL—Poland, SWE—Sweden. Numbered regions: 1—Kaliningrad Region, 2—Pskov Region, 3—Novgorod Region, 4—Leningrad Region, 5—St. Petersburg, 6—the Republic of Karelia, 7—Murmansk Region, 8—Vologda Region, 9—Arkhangelsk Region, 10—the Komi Republic, 11—Nenets Autonomous Okrug. Note: tick collection was not performed in regions 7, 10, or 11.

SFG Rickettsiae prevalence varied according to collection locality. High prevalence levels were identified in the Kaliningrad Region, Pskov Region, Novgorod Region, and in St. Petersburg. In contrast, low levels were detected in the Leningrad Region, the Republic of Karelia, Vologda Region, and in the Arkhangelsk Region. Data on SFG Rickettsiae prevalence in ticks from Northwest Russia’s administrative regions are shown in Figure 2.

The administrative regions with an SFG Rickettsiae prevalence of above 13.6% included: Guryevsky and Zelenogradsky districts of the Kaliningrad Region; Gatchinsky and Kingiseppsky districts of the Leningrad Region; Novgorodsky district of the Novgorod Region; Pechorsky and Sebezhsky districts of the Pskov Region; and Kurortny district of St. Petersburg.

The administrative regions with an SFG Rickettsiae prevalence of 11.7–13.6% included Vsevolozhsky and Priozersky districts of the Leningrad Region; Valdaysky district of the Novgorod Region; and the Nevelsky, Palkinsky, and Pustoshkinsky districts of the Pskov Region.

The administrative regions with an SFG Rickettsiae prevalence below 11.7% were: Onezhsky, Pinezhsky, and Shenkursky districts, as well as the cities of Arkhangelsk, Novodvinsk, and Severodvinsk in the Arkhangelsk Region; Vyborgsky, Kirovsky, Lodeynopolsky, Lomonosovsky, and Luzhsky districts of the Leningrad Region; Okulovsky and Shimsky districts of the Novgorod Region; Ostrovsky, Porkhovsky, and Strugo-Krasnensky districts, as well as the Pskov Urban Okrug, in the Pskov Region; Kondopozhsky district and Petrozavodsk Urban Okrug in the Republic of Karelia; the Primorsky district of St. Petersburg; as well as the Vytegorsky, Gryazovetsky, and Cherepovetsky districts of the Vologda Region.

Based on analysis of the obtained *gltA* gene fragment sequences, circulation of five SFG Rickettsiae species was detected within the study area (Table 2): *R. helvetica* (88%), *R. conorii* subsp. *raoultii* (9.8%), *Candidatus* R. tarasevichiae (0.9%), *R. monacensis* (0.9%), and *R. felis* (0.4%). Specifically, the Kaliningrad Region was characterized by the circulation of *R. helvetica* and *R. conorii* subsp. *raoultii*; Leningrad Region by *R. helvetica* and R. *felis*; Novgorod Region by *R. helvetica*, *R. conorii* subsp. *raoultii*, and *Candidatus* R. tarasevichiae; and Pskov Region by *R. helvetica* and *R. monacensis*. The Vologda Region, the Republic of Karelia, and St. Petersburg were characterized solely by the circulation of *R. helvetica*. Coinfection with *Candidatus* R. tarasevichiae and *R. helvetica* was detected in two female *I. persulcatus* specimens collected in Novgorod Region.

**Figure 3:**
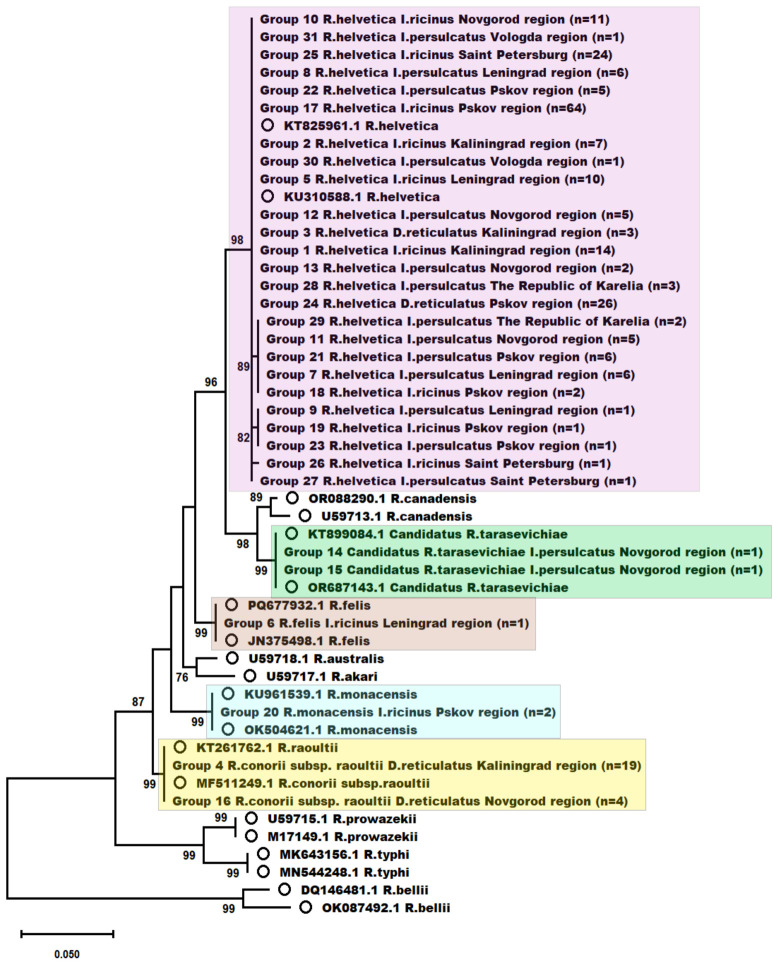
Phylogenetic tree reconstructed by the maximum likelihood method based on analysis of *gltA* gene fragment sequences (alignment length: 757 bp). The tree was constructed using the T93+G nucleotide substitution model. Branch support was assessed by bootstrap analysis with 1000 replicates; bootstrap support values (>70%) are indicated at the respective nodes. Sequences generated in this study are highlighted in distinct colors. Reference sequences retrieved from the NCBI GenBank database are denoted by circles.

Phylogenetic analysis of the detected SFG Rickettsiae, conducted based on *gltA* gene fragment sequences from 236 isolates, demonstrated that all obtained isolates clustered reliably with the reference sequences of the corresponding SFG Rickettsiae representatives (Figure 3). Bootstrap support values at the nodes clustering the sequences obtained in this study with the reference sequences exceeded 70%, thereby confirming the robustness of species identification.

## 4. Discussion

Current data on the species structure of SFG Rickettsiae and their prevalence in Northwest Russia are virtually absent. The existing studies on the epidemiology of tick-borne rickettsioses were conducted a long time ago and do not fully reflect the current state of the problem, or are limited to small geographic areas. Thus, the results obtained in this large-scale study characterize these aspects for the first time. The identification of SFG Rickettsiae species circulating in ticks in a given region and capable of causing human disease is essential for refining the clinical characterization of tick-borne rickettsioses and optimizing patient treatment.

Ixodid ticks act not only as vectors, but also as key reservoirs of *Rickettsia* in natural foci. Animals and birds also constitute important components in the epidemiology of SFG rickettsioses. They serve both as reservoir hosts for *Rickettsia* spp. SFG members and as hosts for feeding ticks. In addition, they participate in horizontal transmission of the pathogen by enabling naive ticks to acquire infection during blood meals on infected animals. Such birds and other animals may facilitate the long-distance dispersal of infected ticks. According to a systematic review, SFG Rickettsiae have been identified in 43 wild mammal species, most frequently Rodentia, Chiroptera, and Carnivora [1]. They have also been found in seven domestic animal species, predominantly dogs, camels, and cattle. Wild mammals may act as sentinels of SFG Rickettsiae circulation in natural foci. In accordance with the One Health concept, serological and molecular genetic data from wild and domestic mammals are important for assessing the activity of natural foci and predicting the risk of human infection [15]. However, tick surveillance remains the priority insofar as it is the most effective and informative approach.

Our findings confirm that Northwest Russia lies within the sympatric zone of three epidemiologically significant ixodid tick species: *I. ricinus*, *I. persulcatus*, and *D. reticulatus* [10,11]. This indicates a potentially high species diversity of SFG Rickettsiae circulating in this region and a considerable public health risk associated with the infections they cause.

The efficiency of ticks as vectors is determined by their prolonged feeding duration and intensive salivation during this period [16]. The duration of blood feeding in females varies over a wide range (from 4 to 22 days), whereas males remain attached to the host for periods ranging from 5 min to 2 h. Accordingly, the primary role in human infection is attributed to females, which attach for extended periods [17]. Moreover, findings from systematic reviews [18,19,20] indicate that female ticks attach to humans more frequently than males. Taken together with the data obtained in this study demonstrating a higher prevalence among female ticks, and combined with their capacity for transovarial transmission of SFG Rickettsiae [7,8,9], this underscores their greater epidemiological significance compared with males.

The frequency of SFG Rickettsiae DNA detection may depend on tick species [21]. Specifically, the more frequent detection of SFG Rickettsiae DNA in *D. reticulatus* compared with *I. ricinus* and *I. persulcatus*, as well as in *I. ricinus* compared with *I. persulcatus*, observed in this study is broadly consistent with data previously reported by several investigators [11,22,23,24,25].

The prevalence values obtained in this study for certain regions differ markedly from those previously reported. Specifically, the SFG Rickettsiae prevalence in ticks collected in Kaliningrad Region reported in a prior study was 11.5% [11], compared with 39.6% in this study. Similarly, previously reported prevalence values were 1.6% for Novgorod Region [26] (19.3% herein), 7.5% for Arkhangelsk Region [26] (0.5% herein), up to 7.4% for Vologda Region [27] (2.4% herein), and 20.4% for the Republic of Karelia [28] (3.0% herein). Such considerable discrepancies may be attributed to differences in tick collection sites, the species and sex composition of the tick samples, the number of ticks examined, detection methods, and temporal variations in the activity of natural foci. To our knowledge, this is the first report of SFG Rickettsiae prevalence in ixodid ticks collected in the Leningrad Region, Pskov Region, and St. Petersburg.

The overall SFG Rickettsiae prevalence in ticks detected in this study was 12.6%, which differs from the results of investigations conducted in several countries of the Baltic region. For instance, in Finland, the prevalence of SFG Rickettsiae reported in various studies reached up to 5.1% [29]; in Denmark and Norway, up to 5.8% [22]; and in Sweden, up to 9.6% [30]. In contrast, higher prevalence rates have been documented in Germany (64.0%), Latvia (19.5%), and Estonia (13.5%) [31,32,33]. Such discrepancies may be attributed to differences in the tick species composition of the samples, the number of ticks examined, and variations in the level of natural foci activity across different regions. For example, the study by Kohn et al. [31] examined only *D. reticulatus* ticks. The higher prevalence of SFG Rickettsiae observed in our study compared to some other investigations may also be explained by our use of a screening test as the sole method for prevalence estimation.

A well-established and effective scheme for the detection and species identification of SFG Rickettsiae has been proposed by Fournier et al. [34]. However, various modifications of this scheme are also used based on species typing using two genes, or even one gene [24,29,32,35,36]. These can also effectively discriminate between SFG Rickettsiae species. Undoubtedly, increasing the number of targets enhances the discriminatory power and informativeness of the methodological approach. Nevertheless, the use of a shorter scheme has also proven to be effective.

In this study, we report the circulation of five SFG Rickettsiae species in natural foci of Northwest Russia: *R. helvetica*, *R. conorii* subsp. *raoultii*, *Candidatus* R. tarasevichiae, *R. monacensis*, and *R. felis*. Although *R. monacensis* has been repeatedly detected in ticks collected in neighboring countries, including Latvia, Poland, Finland, and Estonia [24,29,32,37], this study reports its first detection in Northwest Russia. This finding was confirmed by the identification of this species in two specimens and by sequencing fragments of three genes (*gltA*, *ompA*, *ompB*). Furthermore, to our knowledge, this is the first report of *R. felis* detection in questing ixodid ticks in Russia. This finding was confirmed by the identification of this species in one specimen and by sequencing fragments of two genes (*gltA*, *ompB*).

We also report, for the first time, the widespread distribution and dominance of *R. helvetica* in natural foci of Northwest Russia. Previously, this species had been detected only in ticks collected in the Komi Republic, Vologda Region, and Kaliningrad Region [11,38,39]. The circulation of *R. helvetica* was detected for the first time in the Leningrad Region, Novgorod Region, Pskov Region, St. Petersburg, and the Republic of Karelia. *R. conorii* subsp. *raoultii* was detected for the first time in the Novgorod Region.

The dominance of *R. helvetica* in the study area prompted us to investigate the genetic heterogeneity within this species. The high amplification and sequencing efficiency of the *gltA* target enabled us to do so through sequence analysis. The results of phylogenetic analysis conducted on the obtained *gltA* gene fragment sequences revealed a certain degree of phylogenetic heterogeneity within the *R. helvetica* cluster: the isolates were distributed across several sub-branches. Nevertheless, all isolates remained within a single monophyletic cluster together with the corresponding reference sequences, confirming their affiliation with this species. The observed moderate intraspecific diversity likely reflects natural genetic variability of this gene in *R. helvetica*. In contrast, the clusters of *R. conorii* subsp. *raoultii*, *Candidatus* R. tarasevichiae, and *R. monacensis* exhibited phylogenetic homogeneity, which may be attributed to the limited sample size. The phylogenetic distances corroborate the obtained data regarding the species-level assignment of the detected SFG Rickettsiae.

The *gltA* gene is widely employed for species identification of SFG Rickettsiae, including the analysis of its sequences by phylogenetic methods [24,32]. According to our data, its discriminatory power does not permit the differentiation of isolates based on geographic origin or tick host species. However, it enables the most effective species-level identification among *Rickettsia* spp. SFG.

In addition to the predominantly detected monoinfection in ticks, coinfection with *R. helvetica* and *Candidatus* R. tarasevichiae was detected in this study in two female *I. persulcatus* specimens collected in the Novgorod Region. A systematic review on the global distribution of SFG Rickettsiae [1] previously reported the detection of *Candidatus* R. tarasevichiae coinfection with *R. heilongjiangensis* in *Haemaphysalis concinna* ticks and with *R. conorii* subsp. *raoultii* in *Dermacentor silvarum* and *Haemaphysalis concinna* ticks, as well as *R. helvetica* coinfection with *R. monacensis* in *Ixodes ventalloi* and with *R. slovaca* in *D. reticulatus*. Thus, our findings expand current knowledge regarding the possible combinations of SFG Rickettsiae species in coinfected ticks. Following the bite of a coinfected tick to a human host, a mixed infection caused by two distinct SFG Rickettsiae species may develop, potentially increasing the likelihood of clinically manifest disease and leading to an atypical and/or more severe clinical course [40,41].

Although five SFG Rickettsiae species were identified in the surveyed region, the actual species diversity may be higher. Based on data compiled in a systematic review [1], SFG Rickettsiae representatives were grouped into five clusters according to their spatial distribution and ecological niche. According to this classification, the SFG Rickettsiae species detected in this study belong to Clusters I and IV, which also include other SFG Rickettsiae species, such as *R. slovaca* and *R. sibirica*. This may suggest that these *Rickettsia* species could also circulate in natural foci of Northwest Russia.

Despite using a classical study design in the first stage of our work, we encountered certain limitations and consequently adopted a shorter scheme. This may have introduced some drawbacks, namely a potential reduction in informativeness due to the decreased number of targets used (1–3 per sequenced sample). Our shift in strategy was prompted by two factors. The first was low amplification efficiency for certain genes. The second was low DNA concentration in certain samples (Ct > 35 in real-time PCR for a substantial number of samples). Nevertheless, the use of a reduced set of targets for *Rickettsia* species typing effectively allowed us to characterize species diversity. This approach enabled us to characterize the species structure of the SFG Rickettsiae population in ticks collected in Northwest Russia. The new data not only significantly surpass previous studies in terms of sample size and geographic coverage, but also, in many cases, in terms of the actual findings.

## 5. Conclusions

The findings obtained indicate the existence of natural foci of tick-borne rickettsioses in Northwest Russia and demonstrate considerable species diversity of SFG Rickettsiae within these foci. The results of this study confirm the circulation of five SFG Rickettsiae species: *R. helvetica*, *R. conorii* subsp. *raoultii*, *Candidatus* R. tarasevichiae, *R. monacensis*, and *R. felis*. The presence of *R. monacensis* in ixodid ticks collected within the study area was detected for the first time, and *R. felis* was identified in Russia for the first time. We report an overall SFG Rickettsiae prevalence of 12.6% in ixodid ticks collected from vegetation in Northwest Russia.

The role of the pathogens detected in this study in the infectious disease burden among the population residing in Northwest Russia remains incompletely understood insofar as, despite their established pathogenicity, cases of tick-borne rickettsioses are rarely reported in the study area. This likely reflects substantial underdiagnosis of this disease group. Many researchers anticipate that this group of microorganisms will continue to evolve. This highlights the necessity for rigorous surveillance of SFG Rickettsiae distribution based on data derived from field studies of pathogen vectors and reservoirs.

## Figures and Tables

**Table 1:** Ticks collected and screening results for the presence of SFG Rickettsiae DNA.

Characteristics		Number of Ticks Collected	Prevalence of SFG Rickettsiae, %	95% CI
Tick species	*I. ricinus*	1683	22.5	20.5–24.5
	*I. persulcatus*	2404	3.0	2.4–3.8
	*D. reticulatus*	479	26.3	22.4–30.5
Tick sex	Male	2268	10.5	9.3–11.8
	Female	2298	14.8	13.3–16.3
Collection site	Arkhangelsk Region	406	0.5	0.06–1.8
	Kaliningrad Region	192	39.6	32.6–46.9
	Leningrad Region	1534	7.8	6.5–9.2
	Novgorod Region	218	19.3	14.3–25.1
	Pskov Region	1076	22.5	20.0–25.1
	Republic of Karelia	267	3.0	1.3–5.8
	St. Petersburg	583	13.9	11.2–17.0
	Vologda Region	290	2.4	1.0–4.9
Total:		4566	12.6	11.7–13.6

**Table 2:** SFG Rickettsiae detected in various tick species by gene target.

Tick Species	SFG Rickettsiae Species	Number of Nucleotide Sequences for Gene Fragment, *n*			
		*gltA*	*ompA*	*ompB*	*sca4* (Gene D)
*I. ricinus*	*R. helvetica*	134	-	37	39
	*R. monacensis*	2	2	2	-
	*R. felis*	1	-	1	-
*I. persulcatus*	*R. helvetica*	45	-	12	9
	*Candidatus* R. tarasevichiae	2	-	-	-
*D. reticulatus*	*R. helvetica*	29	-	2	-
	*R. conorii* subsp. *raoultii*	23	21	23	11
Total:		236	23	77	59

Note: The accession numbers of the various gene fragment sequences obtained in this study are listed in Table A3.

## Data Availability

The original contributions presented in this study are included in the article. Further inquiries can be directed to the corresponding author.

## Abbreviations

The following abbreviations are used in this manuscript:

AIC	Akaike Information Criterion
BG	Bellii group
BLAST	Basic Local Alignment Search Tool
BLR	Belarus
CG	Canadensis group
CI	Confidence interval
DNA	Deoxyribonucleic acid
EST	Estonia
FIN	Finland
LTU	Lithuania
LVA	Latvia
ML	Maximum Likelihood
NA	Nucleic acid
NCBI	National Center for Biotechnology Information
NOR	Norway
OR	Odds ratio
PBS	Phosphate-buffered saline
PCR	Polymerase chain reaction
POL	Poland
RNA	Ribonucleic acid
SFG	Spotted Fever Group
SWE	Sweden
TBE	Tris-borate-EDTA buffer
TG	Typhus group

## Appendix A

**Table A1:** Oligonucleotide primers used for PCR amplification and Sanger sequencing.

Gene	Primer	Amplicon Size (bp)	Gene Position	Annealing Temperature (°C)	Reference
*gltA*	CS409f CCTATGGCTATTATGCTTGC RP1258r ATTGCAAAAAGTACAGTGAACA	770	409–428 1178–1157	56 °C	[42]
*ompA*	190.70f ATGGCGAATATTTCTCCAAAA 190.701r GTTCCGTTAATGGCAGCATCT	632	70–90 701–681	60 °C	[43]
*ompB*	120–2788f AAACAATAATCAAGGTACTGT 120–3599r TACTTCCGGTTACAGCAAAGT	812	2788–2808 3599–3579	57 °C	[44]
*sca4* (gene D)	D1f ATGAGTAAAGACGGTAACCT D928r AAGCTATTGCGTCATCTCCG	928	1–20 928–907	58 °C	[45]

**Table A2:** Reference sequences from the NCBI GenBank database used for comparative analysis.

Microorganism	GenBank Accession Number	Isolate/Strain/Clone Name	Geographic Location Name
*R. helvetica*	KT825961	clone 185Skh	Far East, Sakhalin Island, Russia
	KU310588	isolate Novosibirsk-08-5	Novosibirsk region, Russia
*R. conorii* subsp. *raoultii*	KT261762	strain Alashankou-97	China
	MF511249	isolate M197	-
*Candidatus* R. tarasevichiae	KT899084	isolate Hc9	-
	OR687143	isolate Tuva-4	Republic of Tuva, Russia
*R. monacensis*	KU961539	isolate Crimea-3 type II	-
	OK504621	isolate PoTiR20_2021	Portugal
*R. felis*	PQ677932	isolate S5jing	China
	JN375498	isolate LI16	Barra Mansa, Medio Paraiba, Rio de Janeiro, Brazil
*R. australis*	U59718	strain Phillips	-
*R. akari*	U59717	strain MK[Kaplan]	-
*R. prowazekii*	U59715	strain Breinl	-
	M17149	clone pMW264	-
*R. typhi*	MK643156	strain InDRE 65-19	Mexico
	MN544248	isolate CFDVUAM001	Mexico
*R. bellii*	DQ146481	isolate Ixodes	-
	OK087492	isolate BJYW33	China
*R. canadensis*	OR088290	isolate Laiyuan-Hl-120	Laiyuan county of Hebei, China
	U59713	strain 2678	-

Note: “-” indicates that data are not available in the NCBI GenBank database.

**Table A3:** NCBI GenBank database accession numbers of the sequences generated in this study.

SFG Rickettsiae Species	Gene			
	*gltA* (*n* = 236)	*ompA* (*n* = 23)	*ompB* (*n* = 77)	*sca4* (Gene D) (*n* = 59)
*R. helvetica*	PV595500–PV595599, PV632026–PV632082, PX532154, PX532163–PX532168, PX532171–PX532179, PZ053707–PZ053715, PZ053720–PZ053723, PZ053725–PZ053727, PZ053729–PZ053733, PZ173663–PZ173675, PZ173677	-	PX532180–PX532183, PZ053637, PZ053642–PZ053654, PZ053673–PZ053692, PZ173678–PZ173689, PZ173691	PZ230382–PZ230393, PZ230396–PZ230403, PZ230406, PZ230414–PZ230440
*R. conorii* subsp. *raoultii*	PX532145–PX532153, PX532155–PX532162, PX532169, PX532170, PZ053716–PZ053719	PZ053694–PZ053706, PZ173693–PZ173700	PZ053638–PZ053641, PZ053655–PZ053672, PZ053693	PZ230394, PZ230395, PZ230404, PZ230405, PZ230407–PZ230413
*Candidatus* R. tarasevichiae	PZ053724, PZ053728	-	-	-
*R. monacensis*	PV632083, PZ173677	PX532186, PZ173692	PX532184, PZ173690	-
*R. felis*	PV632084	-	PX532185	-
